# Effect of Phacoemulsification on Sub Foveal Choroidal and Central Macular Thickness as measured by Swept Source Optical Coherence Tomography

**DOI:** 10.12669/pjms.39.4.6322

**Published:** 2023

**Authors:** Javeria Ahmed, Ahsan Mukhtar, Mohammad Asim Mehboob, Asfandyar Khan

**Affiliations:** 1 Dr. Javeria Ahmed, MBBS Armed Forces Institute of Ophthalmology, Rawalpindi, Pakistan; 2 Dr. Ahsan Mukhtar, MBBS, FCPS, FCPS VRO, FRCS Armed Forces Institute of Ophthalmology, Rawalpindi, Pakistan; 3 Dr. Mohammad Asim Mehboob, MBBS, FCPS, FRCS, FRCSED, FICO, CHPE Armed Forces Institute of Ophthalmology, Rawalpindi, Pakistan; 4 Dr. Asfandyar Khan, MBBS, FCPS, FCPS VRO, MRCS Armed Forces Institute of Ophthalmology, Rawalpindi, Pakistan

**Keywords:** Cataract Surgery, Central macular thickness, Subfoveal choroidal thickness, Swept source OCT

## Abstract

**Objectives:**

To study the effect of Phacoemulsification on Sub Foveal Choroidal Thickness (SFCT) and Central Macular Thickness (CMT) as measured by Swept Source Optical Coherence Tomography (OCT).

**Methods:**

This experimental study was conducted at Armed Forces Institute of Ophthalmology (AFIO), Rawalpindi from April 2021 to February 2022. One hundred eyes of 100 patients with age related cataract underwent uneventful phacoemulsification surgery. Pre-operative SFCT and CMT was measured and compared with SFCT and CMT at one week, one month and three months after surgery using swept source OCT.

**Results:**

Mean age of study population was 56.76±8.31 years. Out of 100 patients, 46 (46%) were males and 54 (54%) were females. Mean pre-operative CMT, one week, one month and three months post-operative CMT was 233.95±9.46 μm, 232.88±8.59 μm, 230.38±10.62 μm and 230.67±7.55 μm respectively. Mean pre-operative SFCT, one week, one month and three months post-operative SFCT was 337.14±8.41 μm, 339.14±9.63 μm, 339.39±11.96 μm and 351.39±9.19 μm respectively. The difference of mean change in CMT from baseline at one week, one month and three months post-operatively was not statistically significant. The difference of mean change in SFCT from baseline at one week and one month post-operatively was not statistically significant. However, the difference of mean change in SFCT from baseline at three months post-operatively was statistically significant (p<0.05).

**Conclusion:**

Uneventful phacoemulsification surgery does not have any effect on central macular thickness, however there is a significant increase in subfoveal choroidal thickness at three months after surgery.

## INTRODUCTION

Age related cataract formation is one of the leading causes of reversible blindness in the world.^1^ The burden of age related cataract is specifically high in resources limited countries, where it affects quality of life of millions of people living without basic health care facilities.^1^ With evolution of fine surgical instrumentation and microscopic visualization, cataract surgery has been revolutionized. It is one of the commonest performed surgical procedures in the world, with reasonable safety and efficacy profile.^2^ Like all surgical procedures, cataract surgery has effect on different anatomical features of human eye. Many changes have been reported in human eye after routine phacoemulsification cataract surgery.^3^ While some of the changes have a profound effect on the vision and vision related quality of life, the benefits of surgery cannot be undermined.^4^

Swept source Optical Coherence Tomography (OCT) is a recent addition to marvels of ophthalmic tissue assessment. It is a non-invasive testing modality, which allows in-vivo careful examination of histological layers of retina and choroid with meticulous precision and accuracy.^5^ Impact of different diseases on retinal and choroidal tissue has been assessed using swept source OCT.^5^ This has led to development of treatment protocols being used for improvement in vision related quality of life in patients.

OCT has been used to assess changes in retina after phacoemulsification procedure. Few studies have evaluated role of phacoemulsification on Central Macular Thickness (CMT).^6^ Any change or oedema after cataract surgery has profound effect on vision of patient undergoing cataract surgery. Different studies have also evaluated the role of uneventful phacoemulsification cataract surgery on Subfoveal Choroidal Thickness (SFCT).^7^ Another study conducted on Pakistani eyes reported increase in SFCT one month after cataract surgery.^8^ Aim of this study was to study the mean change in SFCT and CMT from baseline, at one week, one month and three months after uneventful phacoemulsification cataract surgery as measured by Swept Source OCT.

## METHODS

A total of 100 eyes of 100 patients was enrolled for study. This study had prior approval from ethical review committee of Armed Forces Institute of Ophthalmology, Rawalpindi (approval vide certificate 256/ERC/AFIO dated 23 April 2021). All patients gave written informed consent prior to enrolment in study. Sample size of 100 eyes was estimated after keeping significance level of 5% and power of test at 80%.^9^ Patients from either gender, with significant age related cataract warranting surgical removal, aged from 40-60 years were included in the study. Patients with per-operative complications, prolonged surgery, and prior retinal disease, history of uveitis, macular oedema, trauma, glaucoma, ocular laser or injections treatment were excluded. Subjects fulfilling the inclusion criteria were subjected to assessment of visual acuity, best corrected visual acuity, cataract grading according to lens opacity classification system,^10^ and detailed fundoscopy. Patients then underwent optical intraocular lens power calculation and pre-operative CMT and SFCT was measured using Topcon DRI OCT Triton (Topcon Healthcare, USA). All patients then underwent routine phacoemulsification cataract surgery by a single experienced surgeon. Post-operatively, all patients received standard post-operative topical medication (Moxifloxacin, 2%, four times daily and Prednisolone, 0.1%, four hourly) for two weeks. The patients underwent routine follow up at one week, two weeks, one month and three months post operatively and were subjected to visual acuity measurement and measurement of CMT and SFCT by Topcon DRI OCT Triton (Topcon Healthcare, USA). Patients with complicated cataract surgery, or post-operative inflammation or macular oedema were excluded from the study. The pre devised proforma was completed endorsing subject’s demography, ocular examination and investigations findings.

### Statistical Analysis:

Data was evaluated and analyzed using Statistical Program for Social Sciences (SPSS) version 17. Mean and standard deviation was reported for continuous variables (Age, CMT, SFCT) while frequency and percentage for nominal data (gender, laterality of eyes). Shapiro Wilk’s test was used to check normality of data. Post normality testing, Paired t-test was used to compare post-operative values at one week, one month and three months from pre-operative values. A p-value of ≤0.05 was considered statistically significant.

## RESULTS

Initially, a total of 110 patients were included in the study. After excluding complicated surgery cases, and post-operative inflammation cases, a final of 100 eyes were included. A total of 100 eyes of 100 patients completing the follow up were thus included and analysed. Demographic details, mean pre-operative CMT, SFCT, along with one week, one month and three months post-operative CMT and SFCT are given in Table-I. Comparison of post-operative CMT and SFCT from pre-operative value is given in Table-II. The difference of mean change in CMT from baseline at one week, one month and three months post-operatively was not statistically significant. The difference of mean change in SFCT from baseline at one week and one month post-operatively was not statistically significant. However, the difference of mean change in SFCT from baseline at three months post-operatively was statistically significant (p<0.05).

**Table-I:** Demography and Clinical Data of Study Population (n=100).

Variable	Study Population (n=100)
Age (Years) Mean ± SD	56.76±8.31
Gender	46 /54
(Male/Female)	(46%)/ (54%)
Laterality	52 / 48
Right/Left	(52%)/(48%)
Pre-Operative CMT (μm) Mean ± SD	233.95±9.46
Oneweek Post-Operative CMT(μm) Mean ± SD	232.88±8.59
One month Post-Operative CMT(μm) Mean ± SD	230.38±10.62
Three months Post-Operative CMT(μm) Mean ± SD	230.67±7.55
Pre-Operative SFCT (μm) Mean ± SD	237.14±8.41
One week Post-Operative SFCT (μm) Mean ± SD	239.14±9.63
One month Post-Operative SFCT (μm) Mean ± SD	239.39±11.96
Three months Post-Operative SFCT (μm) Mean ± SD	251.39±9.19

**Table-II:** Comparison of Post-operative CMT and SFCT from Pre-Operative Value in Study population (n=100).

Variable	Measurements CMT (μm) (n=100)	Measurements SFCT (μm) (n=100)
***One week Follow up***		
Pre-operative	233.95±9.46	237.14±8.41
One week post-operative	232.88±8.59	239.14±9.63
p Value*	0.875	0.342
***One month Follow up***		
Pre-operative	233.95±9.46	237.14±8.41
One month post-operative	230.38±10.62	239.39±11.96
p Value*	0.673	0.547
***Three months Follow up***		
Pre-operative	233.95±9.46	237.14±8.41
Three months post-operative	230.67±7.55	251.39±9.19
p Value*	0.521	0.012

* Paired ‘t’ Test.

## DISCUSSION

There are different reasons hypothesized for change in CMT and SFCT after phacoemulsification cataract surgery. It is believed that even after uneventful cataract surgery, subclinical changes in the retinal layers occur due to the release of inflammatory mediators which not only affect the retina but also the choroid. It is thought that the ultrasound generated currents in vitreous and retina due to phacoemulsification lead to the release of inflammatory mediators particularly prostaglandins which has role in increasing central macular thickness.^11^ This release of prostaglandins is expected to be transient and after some period, the quantity is again reduced, bringing CMT to baseline levels. Pasova Petra et al found increase in CMT to be temporary in nature, with return to baseline after six months.^12^ In our study, there was no statistically significant change in CMT even at one week, one month and three months post-operatively. This has been partially attributed to use of Nepafenac eye drops in each case post operatively.^13^ After approval of nepafenac in 2005, it has been widely used for reducing pain, inflammation and decreasing central macular thickness.^13^ Authors believe that topical nepafenac has a protective role in prevention of change in CMT in our case.

SFCT has also been implicated to change in much retinal vascular pathology like diabetes and retinal vein occlusion. Many studies have concluded the role of non-steroidal anti-inflammatory drugs and corticosteroids in reducing the SFCT and other complications.^14^,^15^ Ibrahim et al concluded that SFCT increases up to 03 months duration.^16^ In our study, there was no statistically significant change at one week and one month post operatively, but there was significant change in SFCT from baseline at three months post-operatively. Multiple reasons have been postulated for change in SFCT. It is supposed that surgical trauma impairs the blood aqueous barrier leading to the accumulation of prostaglandins, cytokines and immune complexes. These mediators pass through the anterior segment and then through posterior segment reaching to the retina and choroid leading to impairment of outer and inner blood retinal barrier.^17^ A resultant dilatation in choroidal vessels leads to change in SFCT which can be accurately measured.

In our study we have included patients with uneventful, without complicated surgery and no vitreous loss. It is believed that posterior capsular rupture and resultant vitreous loss can lead to changes in macula. In a study done by Von Jagow B and associates, CMT increased in patients undergoing routine phacoemulsification surgery as measured by OCT.^18^ In another study conducted on eyes undergoing small incision cataract surgery, there was gross difference in CMT from baseline.^19^ It is assumed that small incision cataract surgery induces significant change in vitreous physically which then leads to change in CMT and resultant post- operative inflammation. We didn’t observe any change in CMT, which is probably attributed to uneventful surgeries, non-inclusion of patients with already established retinal vascular diseases and inclusion of topical drugs to control post-operative pain and inflammation. The CMT values of our study are in accordance with the already present international literature.^20^

The authors observed that SFCT was either stable or increased in early post-operative period of one month. However, there were dilated choroidal vessels observed on OCT and validated by change in SFCT three months after surgery. That resulted in increase in SFCT as measured by swept source OCT. There are different postulates for change in choroidal thickness after cataract surgery.^21^-^23^ In one study, it was emphasized that SFCT is increased due to increased choroidal perfusion and decreased intraocular pressure.^21^ Surgical stress leads to increased choroidal perfusion while a decrease in intraocular pressure during surgery leads to permanent changes in subfoveal choroid.^22^ Another study conducted by Bayhan et al also revealed the same results.^23^

We have used Topcon Triton Swept source OCT for measuring the values of CMT and SFCT. Choroidal thickness other than subfoveal choroidal area was not measured as it is believed to partially affect central vision. Since the previous international studies are based on use of spectral domain OCT and fourier domain OCT and not the swept source, it is not able to measure the SFCT with accuracy.^24^

### Limitations:

It includes small sample size, and measurement of subfoveal thickness of choroid only. Further studies with a comparison group, measurement of global and sectorial choroidal thickness and inclusion of patients with retinal and choroidal vascular pathologies will add additional insight to this clinical assessment.

## CONCLUSION

Uneventful phacoemulsification cataract surgery has no impact on central macular thickness in long term, however it causes significant increase in subfoveal thickness of choroid three months after surgery. We propose that patients with clinical conditions involving choroidal and retinal pathologies while undergoing phacoemulsification cataract surgery should be monitored for progression of their posterior segment pathology in order to avoid progression of disease.

### Authors’ Contribution:

**JA:** Conceived, designed manuscript, did data collection, manuscript discussion.

**AM:** Did editing and finally approved manuscript.

**MAM:** Did statistical analysis, manuscript writing, editing.

**AKL**: Did data analysis, critical discussion, Literature review.
